# Characterization of Piezoresistive PEDOT:PSS Pressure Sensors with Inter-Digitated and Cross-Point Electrode Structures

**DOI:** 10.3390/s150100818

**Published:** 2015-01-05

**Authors:** Jer-Chyi Wang, Rajat Subhra Karmakar, Yu-Jen Lu, Chiung-Yin Huang, Kuo-Chen Wei

**Affiliations:** 1 Department of Electronic Engineering, Chang Gung University, Kweishan 33302, Taoyuan, Taiwan; E-Mail: rjtkarmakar@gmail.com; 2 Healthy and Aging Center, Chang Gung University, Kweishan 33302, Taoyuan, Taiwan; 3 Department of Neurosurgery, Chang Gung Memorial Hospital, Kweishan 33305, Taoyuan, Taiwan; E-Mails: alexlu0416@gmail.com (Y.-J.L.); chyinhuang@cgmh.org.tw (C.-Y.H.); kuochenwei@adm.cgmh.org.tw (K.-C.W.)

**Keywords:** piezoresistive, poly(3,4-ethylenedioxythiophene):polystyrene sulfonate (PEDOT:PSS), inter-digitated, cross-point, relaxation time

## Abstract

The piezoresistive characteristics of poly(3,4-ethylenedioxythiophene):polystyrene sulfonate (PEDOT:PSS) pressure sensors with inter-digitated (IDE) and cross-point electrode (CPE) structures have been investigated. A small variation of the resistance of the pressure sensors with IDE without bottom indium-tin-oxide (b-ITO) film and with CPE structures was observed owing to the single carrier-conducting pathway. For the IDE pressure sensors with b-ITO, the piezoresistive characteristics at low and high pressure were similar to those of the pressure sensors with IDE without b-ITO and with CPE structures, respectively, leading to increased piezoresistive pressure sensitivity as the PEDOT:PSS film thickness decreased. A maximum sensitivity of more than 42 kΩ/Pa was achieved. When the normal pressure was applied, the increased number of conducting points or the reduced distance between the PEDOT oligomers within the PEDOT:PSS film resulted in a decrease of the resistance. The piezoresistive pressure sensors with a single carrier-conducting pathway, *i.e.*, IDE without b-ITO and CPE structures, exhibited a small relaxation time and a superior reversible operation, which can be advantageous for fast piezoresistive response applications.

## 1. Introduction

Pressure sensors have a great significance for industrial equipment and they are widely used for the control and monitoring of thousands of applications, such as biomedical, environment, space, and automobiles [1–4]. Many works have been conducted on pressure measurement using various techniques and three major types of pressure sensors have been investigated using capacitive, piezoelectric, and piezoresistive measurements [5–8]. For example, capacitive pressure sensors use a diaphragm and a pressure cavity to create a variable capacitance for detecting strain by applying pressure. Common diaphragm materials are metal, ceramic, and silicon [5,6]. Capacitive pressure sensors have been reported to be suitable for low-pressure measurements because of their sensitive diaphragms, but the linearity of their capacitance is poor due to the parasitic issue during the measurement. For piezoelectric pressure sensors, the piezoelectric effect in certain materials, such as quartz, has been used to measure the strain [5,7]. This technology is commonly employed to measure highly dynamic pressure phenomena but it is insensitive to static responses. Furthermore, piezoresistive pressure sensors have been proposed to detect the strain by applying pressure to change the resistance of test patterns. Materials typically used for piezoresistive pressure sensors are silicon, polysilicon thin films, bonded metal foils, sputtered thin films, and inkjet printing films [5,8–10]. Generally, piezoresistive pressure sensors are the most commonly employed technology in the pressure sensor market owing to their advantages of high sensitivity and low cost.

Conducting polymers have been widely used in organic electronics after their discovery by Shirakawa *et al.* [11,12] because they exhibit promising properties, such as high flexibility, low-cost fabrication process, light weight, and easy tailoring, to obtain the required performance [13,14]. Aside from the above characteristics, some conducting polymers also possess the unique piezoresistive property, which can be used for strain sensor applications [15]. Among conductive materials, poly(3,4-ethylenedioxythiophene) (PEDOT) has gained an outstanding position for pressure sensor applications owing to its high electrochemical and thermal stability, high conductivity, good optical properties, and high transparency [16,17]. To balance the cationic charge of PEDOT and allow the dispersion of PEDOT in water, polystyrene sulfonate (PSS) is introduced to form the water-soluble polymer PEDOT:PSS [17]. PEDOT:PSS can be applied in solid electrolyte capacitors [18], antistatic coatings [19], and anode electrodes in organic electronic devices, such as light-emitting diodes [20], field effect transistors [21], photovoltaic cells [22], and flexible sensors on sensing skin [23]. Moreover, strain sensors with PEDOT:PSS on polyimide and polyethylene terephthalate (PET) flexible substrates have been demonstrated to present sufficient piezoresistive properties for biosensor applications [24–26].

There are two types of electrode patterns being considered for piezoresistive pressure sensors. Inter-digitated electrode (IDE) structures, with feature size in the nanometer scale, are popular in the solid-state physics community and they have been implemented in various devices, including surface acoustic wave (SAW) sensors [27], chemical sensors [28], micro-electro-mechanical systems (MEMS) biosensors [29], and semi-conducting nanowires [30]. The output signal strength of IDEs is controlled through a careful design of the active area, width, and spacing of the electrode fingers. On the other hand, for the cross-point electrode (CPE) structure, the active sensing material is sandwiched between two patterned electrodes. The CPE structure is widely used for emerging memory applications, especially for resistive random access memory (RRAM) devices [31,32]. It has also been used for the high-density strain sensor arrays, where the number of active cells can be increased by increasing the number of top and bottom electrodes [33,34]. However, the effects of IDE and CPE structures on piezoresistive pressure sensors have not yet been understood. In this work, PEDOT:PSS piezoresistive pressure sensors fabricated on a flexible PET substrate with IDE and CPE structures were investigated. The piezoresistive characteristics of these three structures, IDEs with and without b-ITO and a CPE, are strongly dependent on the PEDOT:PSS thickness, which have been examined to be due to the different carrier conducting pathways. Consequently, a specific piezoresistive structure is suggested to achieve a high piezoresistive sensitivity and a small relaxation time with less variation of the resistance, which can be used for future piezoresistive applications.

## 2. Experimental Procedures

### 2.1. Fabrication of the PEDOT:PSS Pressure Sensors

**Figure 1.**
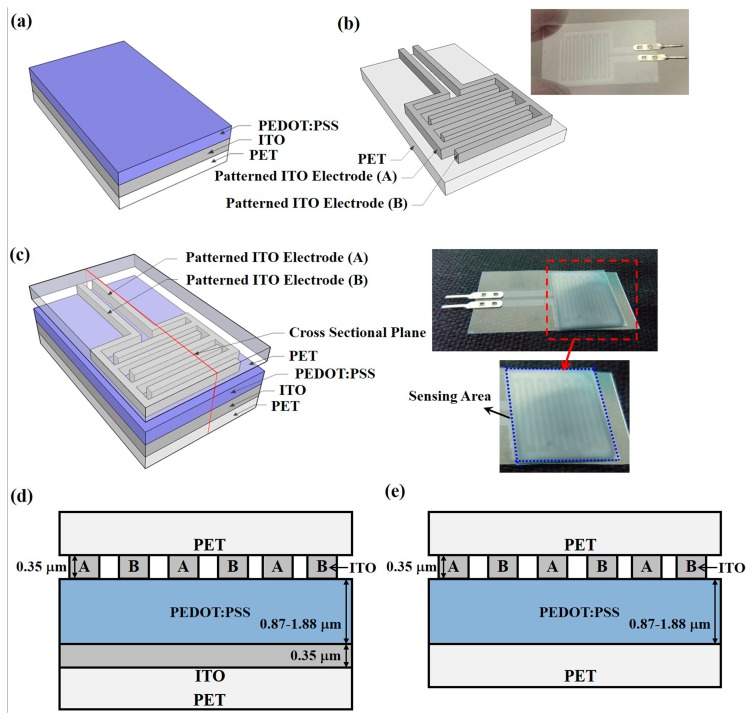
Schematic diagrams of the poly(3,4-ethylenedioxythiophene):polystyrene sulfonate (PEDOT:PSS) pressure sensors with inter-digitated electrode (IDE) structures for (**a**) part I; (**b**) part II; and (**c**) the final device with image picture, and the cross-sectional diagrams of the IDE pressure sensors (**d**) with and (**e**) without b-ITO film.

**Figure 2.**
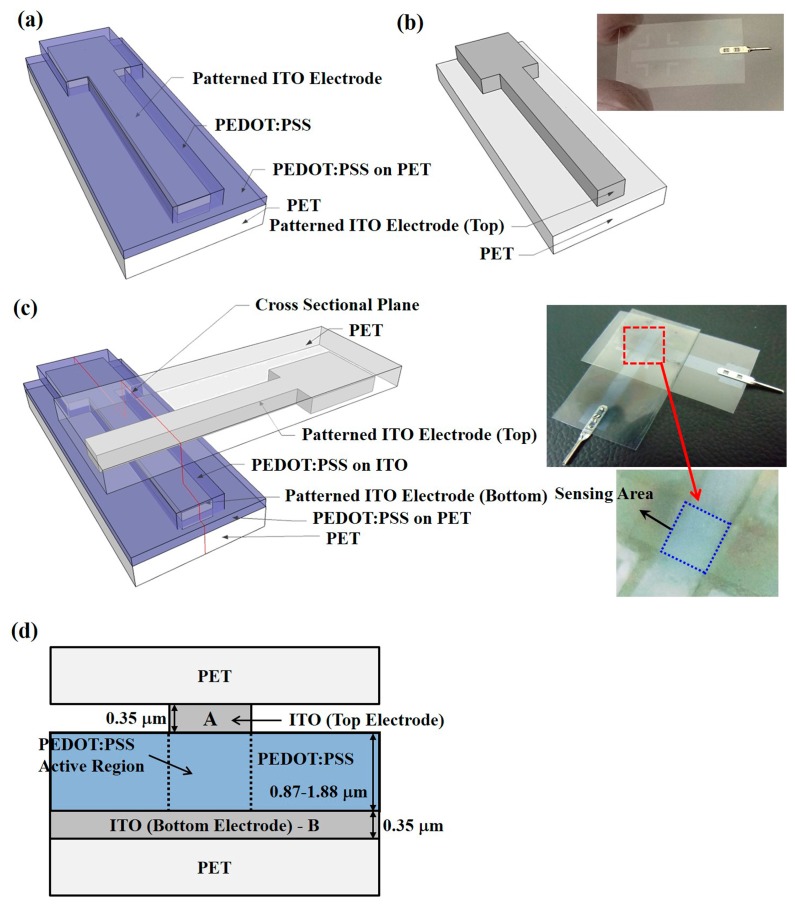
Schematic diagrams of the poly(3,4-ethylenedioxythiophene):polystyrene sulfonate (PEDOT:PSS) pressure sensors with cross-point electrode (CPE) structures for (**a**) part I; (**b**) part II; and (**c**) the final device with image picture; and (**d**) the cross-sectional diagram of the pressure sensors with CPE structures.

The piezoresistive pressure sensors with the PEDOT:PSS conductive material were fabricated on ITO-coated PET substrates. The ITO-coated PET substrates were provided by Win Optical Technology (Taoyuan, Taiwan) and the ITO film was deposited by radio-frequency (RF) sputtering using a roll-to-roll process. ITO with a thickness of 0.35 μm was used as the electrode material, providing the advantages of a high electron density of 10^21^ cm^−3^ in the conduction band and sufficient stability in aqueous solutions for electrochemical applications [35]. Furthermore, ITO also shows transparency to visible light, which enables multiple parameter measurements by using optical and electrical techniques [36]. Two structures, an IDE and a CPE, were prepared, as illustrated in Figures 1 and 2, which show their schematic diagrams, sensor device images, and cross-sectional structures. For part I of the pressure sensors with IDE structures, shown in Figure 1a, the ITO-coated PET substrate was first treated by O_2_ plasma to make the ITO film hydrophilic for the successful coating of PEDOT:PSS films [37]. PEDOT:PSS was spin-coated on the b-ITO films with speeds of 500, 750, and 1000 rpm to obtain an average film thickness of 1.88, 1.32, and 0.87 μm, respectively, measured by an ellipsometer. A PEDOT:PSS solution with a concentration of 1.56 wt. % was synthesized to obtain a PEDOT:PSS film resistivity of 4.85 × 10^7^ Ω-cm by a Hall measurement [38,39]. The use of PEDOT:PSS films with such a high resistivity is due to the need to achieve a suitable piezoresistive response. Subsequently, to make the film dry, all samples were baked at 90 °C for 10 min using a hot plate. For comparison, the PET substrate without b-ITO was also spin-coated with PEDOT:PSS film. On the other hand, for part I of the pressure sensors with CPE structures shown in Figure 2a, the ITO bottom electrode was first patterned using an *aqua regia* solution to obtain the desired patterns. The samples were then treated by O_2_ plasma, spin-coated using the PEDOT:PSS solution, and finally baked. For part II, the ITO electrodes were patterned to form the fingers and top electrodes of the pressure sensors with IDE (Figure 1b) and CPE structures (Figure 2b), respectively. For the sensors with IDE structures, the width and spacing of each finger was 500 μm. Because the width of the top and bottom electrodes was 3000 μm, the active cell area of the sensors with CPE structures was 9 × 10^6^ μm^2^. Figure 1c shows the schematic diagram and image of the final sensor devices with IDE structures, and those of the devices with CPE structures are presented in Figure 2c. From the red cut-line shown in Figures 1c and 2c, the cross-sectional structures of the pressure sensors with IDE with b-ITO and CPE structures were obtained and they are illustrated in Figures 1d and 2d, respectively. In addition, the cross-sectional structure of IDE pressure sensors without b-ITO is also displayed in Figure 1e. All parameters of the piezoresistive pressure sensors with IDE and CPE structures are summarized in Table 1.

### 2.2. Characterization Methodology

After the piezoresistive pressure sensors had been fabricated, their electrical properties were characterized using a Keithley 2450 interactive digital source meter (Keithley Instruments Inc., Cleveland, OH, USA). The samples were placed on a homemade sample holder fabricated by rigid steel and the pressure in vertical direction, also called the normal pressure, was applied using a JSV H1000 vertical stand (ALGOL Instrument Co., Ltd., Taoyuan, Taiwan) equipped with an ALGOL force gauge. A quartz buffer layer of 10^8^ μm^2^, larger than the active area, was used to provide an equal pressure distribution throughout the sensor area. A normal pressure of 0.1 to 20 kPa with a speed of 2 mm/min was applied on the samples to obtain the piezoresistive characteristics at low pressure and identify the carrier conducting pathways of each structure to avoid breaking of quartz plate. Additionally, the reversible testing of the response properties of the pressure sensors was performed with a holding time (*t_h_*) of 10 s.

## 3. Results and Discussion

### 3.1. Piezoresistive Characteristics

**Figure 3.**
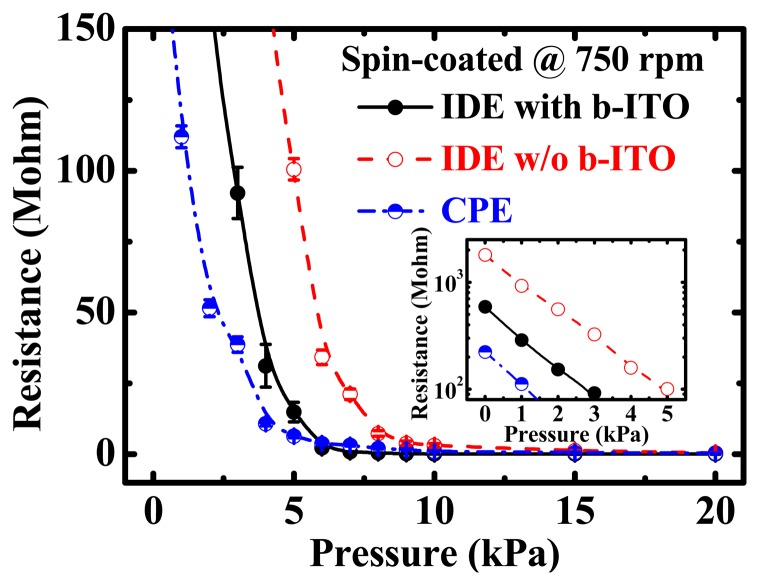
The resistance *versus* pressure (*R-P)* characteristics of the poly(3,4-ethylenedioxythiophene):polystyrene sulfonate (PEDOT:PSS) pressure sensors with inter-digitated electrode (IDE) and cross-point electrode (CPE) structures. The spin speed of 750 rpm was used to coat PEDOT:PSS film and at least 20 samples were measured to obtain the statistical distribution. The inset shows the low-pressure properties of these pressure sensors.

Figure 3 shows the resistance *versus* pressure (*R-P*) characteristics of the PEDOT:PSS pressure sensors with IDE and CPE structures. The low-pressure properties of these sensors are also depicted in the inset figure. A spin speed of 750 rpm was used to coat PEDOT:PSS film on the flexible PET substrates. To obtain the statistical distribution, at least 20 samples were measured for each pressure sensor. As the applied normal pressure increases, the measured resistance decreases, demonstrating the well-known piezoresistive property. It was obtained that the piezoresistive pressure sensitivity of these three samples was approximately 33.73 to 35.66 kΩ/Pa. Compared with IDEs with b-ITO, the pressure sensors with IDEs without b-ITO and with CPE structures presented a smaller variation in the measured resistance. The initial resistance, *i.e.*, the resistance without any pressure, of the pressure sensors with CPE and IDE structures with and without b-ITO was measured to be 263, 597 and 1810 MΩ, respectively. The structure-dependent carrier-conducting pathway is responsible for the different piezoresistive characteristics between the IDE and CPE structures which will be discussed later.

**Figure 4.**
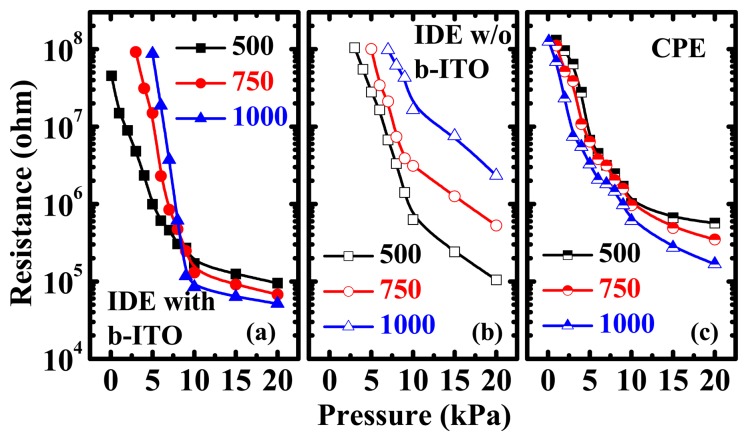
Logarithmic scale of the resistance *versus* pressure (*R-P)* characteristics of the pressure sensors with (**a**) inter-digitated electrode (IDE) with bottom indium-tin-oxide (b-ITO); (**b**) IDE without b-ITO; and (**c**) cross-point electrode (CPE) structures. The characteristics with spin-coating speeds of 500 to 1000 rpm were compared.

To further clarify the carrier conducting mechanism of the examined sensors, the logarithmic scale of the *R-P* characteristics of the PEDOT:PSS pressure sensors with various PEDOT:PSS spin-coating speeds are displayed in Figure 4. It is worth noting that for the IDE pressure sensors with b-ITO, there are two distinct piezoresistive characteristics for the different PEDOT:PSS spin-coating speeds, *i.e.*, the different PEDOT:PSS film thicknesses, at low and high normal pressure (Figure 4a). When the applied pressure is lower than 10 kPa, the measured resistance increases with the spin-coating speed, which is the same trend displayed at all pressures by the IDE sensors without b-ITO, shown in Figure 4b. On the other hand, when the normal pressure is higher than 10 kPa, the measured resistance decreases with the spin-coating speed, which is identical to the characteristics of the CPE sensors at all pressures, shown in Figure 4c. The opposite dependence of PEDOT:PSS film thicknesses on the resistance at some specific normal pressure for the CPE and IDE sensors without b-ITO is observed and the combination of the two aforementioned phenomena is presented in the IDE sensors with b-ITO. Thus, the IDE pressure sensors with b-ITO present an increase of piezoresistive pressure sensitivity when the spin-coating speed increases, as depicted in Figure 4a. The maximum sensitivity of the IDE pressure sensors with b-ITO for a PEDOT:PSS spin-coating speed of 1000 rpm was approximately 42 kΩ/Pa.

### 3.2. Structure-Dependent Conducting Mechanism

**Figure 5.**
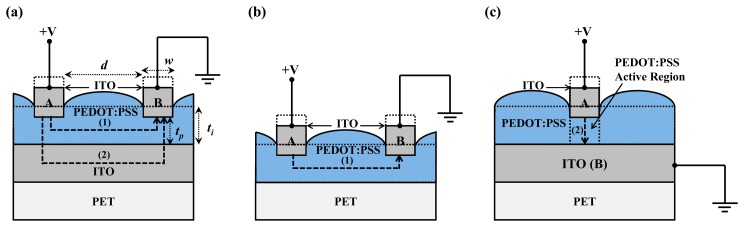
Schematic diagrams of the carrier conducting pathways of the pressure sensors with (**a**) inter-digitated electrode (IDE) with bottom indium-tin-oxide (b-ITO); (**b**) IDE without b-ITO; and (**c**) cross-point electrode (CPE) structures.

Figure 5 illustrates the conducting mechanism of the PEDOT:PSS pressure sensors with IDE and CPE structures. In Figure 5a, there are two conducting pathways of the IDE structure with b-ITO. Paths (1) and (2) represent the horizontal and vertical conduction within the PEDOT:PSS film, respectively. When a low normal pressure is applied on the PEDOT:PSS film, path (1) is the dominant carrier-conducting pathway and the resistance of this path, *R_1_*, increases when the PEDOT:PSS film thickness decreases, *i.e.*, the spin-coating speed increases, as illustrated in Figure 4a. This phenomenon is due to the decrease of the carrier conducting area according to the following equation:

(1) $$R_{1}=\rho \times \frac{d}{A_{t}}$$

where ρ is the resistivity of PEDOT:PSS, *d* is the distance between inter-digitated fingers, and *A_t_* is the cross-sectional area in the horizontal direction, which is related to the thickness of PEDOT:PSS film, *t*. It has been reported that the PEDOT:PSS film can be stretched out under a tensile stress and its length and width are modified accordingly [40]. Therefore, when a high normal pressure is applied to compress the PEDOT:PSS film, the carrier conduction is mainly via path (2). The resistance of path (2), *R_2_*, decreases as the PEDOT:PSS film thickness decreases, which can be ascribed to the following equation:

(2) $$R_{2}=\rho \times \frac{t}{A_{w}}$$

where *t* is the thickness of the PEDOT:PSS film and *A_w_* is the cross-sectional area of the overlapped region between the inter-digitated fingers and the b-ITO film. These characteristics can be further confirmed by the carrier-conducting pathway of the IDE without b-ITO and CPE structures. For the IDE structure without b-ITO shown in Figure 5b, there is only path (1) for the carrier conduction, indicating that the resistance must increase when the PEDOT:PSS film thickness decreases, as shown in Figure 4b. For the initial resistance of the IDE structure without b-ITO, displayed in the inset of Figure 3, the path can be examined by calculating the PEDOT:PSS film resistivity from Equation (1) using *R_1_* = 1810 MΩ, *d* = 500 μm, and *A_t_* = 1.72 = 10^5^ μm^2^. The calculated resistivity of path (1) is 6.22 × 10^7^ Ω-cm, which is nearly the same as the value obtained from the PEDOT:PSS film by the Hall measurement. On the other hand, for the CPE structure in Figure 5c, path (2) is the only conducting pathway. Thus, as the PEDOT:PSS film thickness decreases, the resistance decreases, as presented in Figure 4c. For the initial resistance of the CPE structure in the inset of Figure 3, the path can be confirmed by calculating the PEDOT:PSS film resistivity from Equation (2) using *R_2_* = 263 MΩ, *t* = 1.32 μm, and *A_w_* = 9 × 10^6^ μm^2^. The calculated resistivity of path (2) is 1.79 × 10^11^ Ω-cm, which is three to four orders of magnitude larger than the value obtained for path (1). Nardes *et al.* [41] have proposed that in the horizontal direction of carrier conduction in a PEDOT:PSS film, *i.e.*, path (1) of our structures, the PEDOT-rich lamellas are only separated by the not-completely-closed constrictions, allowing carriers to hop to non-nearest-neighbor sites through a thin or non-existent barrier. On the other hand, in the vertical direction, *i.e.*, path (2) of our structures, the PEDOT-rich domains are separated by thick PSS-lamella barriers, enforcing only nearest-neighbor hopping. Therefore, the resistivity obtained from path (1) is almost the same as the value obtained from the PEDOT:PSS film by the Hall measurement, which is much smaller than that of path (2). With the combination of both vertical and horizontal paths of the IDE sensors with b-ITO, the slope of the *R-P* curves in Figure 4a can be altered by the PEDOT:PSS film thickness, due to the change of dominant carrier conducting pathway within the film.

**Figure 6.**
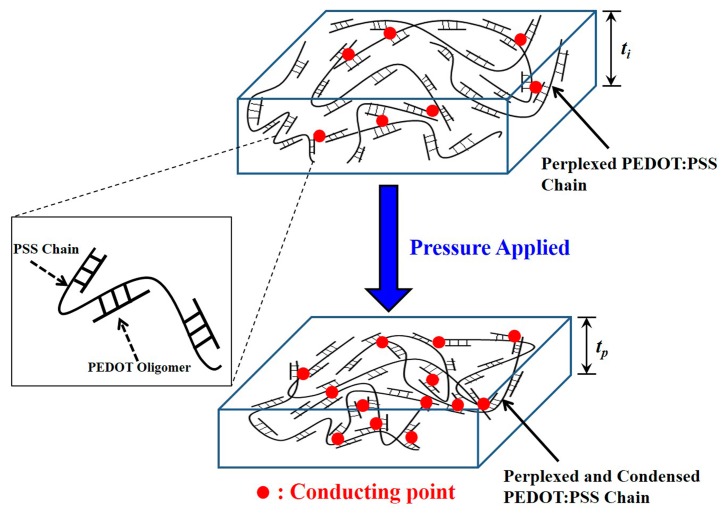
Conducting mechanism of the poly(3,4-ethylenedioxythiophene):polystyrene sulfonate (PEDOT:PSS) chains in the compressed mode. The red points illustrated the conducting points within the PEDOT:PSS film. Perplexed PSS chain with connected PEDOT oligomers was shown in the enlarged figure.

The schematic structures of the PEDOT:PSS films before and after the application of a normal pressure are shown in Figure 6. In this figure, we can observe that the PEDOT oligomers are attached to the long perplexed PSS chain. As we know, the basic conducting mechanism of the PEDOT:PSS film is nearest-neighbor hopping [41,42]. When normal pressure is applied on the film, the perplexed chain of PSS with the attached PEDOTs is condensed from a film thickness of *t_i_* to *t_p_* [40]. Consequently, the possibility of carrier conduction is increased through the nearest-neighbor and non-nearest-neighbor PEDOT grains due to the reduced distance between PEDOT oligomers or the increased number of conducting points, as indicated by the red points in Figure 6. This leads to the decrease of the resistivity of the PEDOT:PSS film. Therefore, all the piezoresistive pressure sensors exhibit a decreased resistance but different trends in the relation of the PEDOT:PSS film thickness with the applied normal pressure, as shown in Figure 4.

### 3.3. Relaxation of Piezoresistive Characteristics with Different Structures

**Figure 7.**
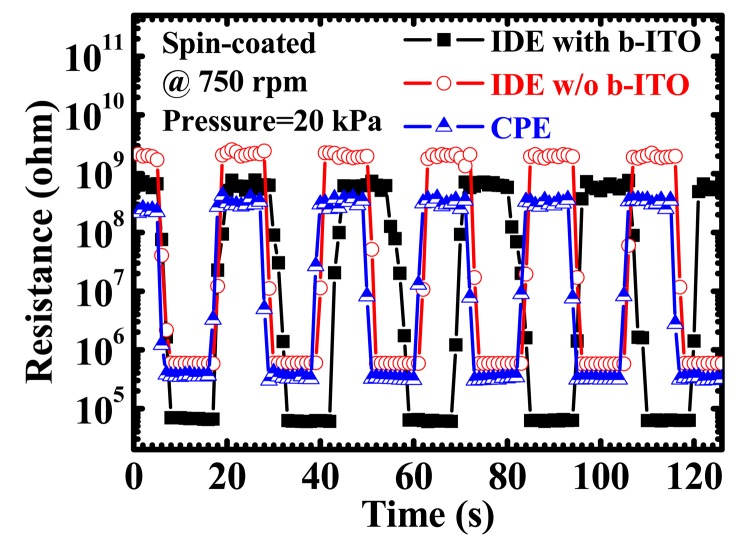
Reversible testing of poly(3,4-ethylenedioxythiophene):polystyrene sulfonate (PEDOT:PSS) pressure sensors with inter-digitated electrode (IDE) and cross-point electrode (CPE) structures for at least five loops. The PEDOT:PSS spin-coating speed of 750 rpm and the applied pressure of 20 kPa under a 10-s holding time were used for the measurement.

**Figure 8.**
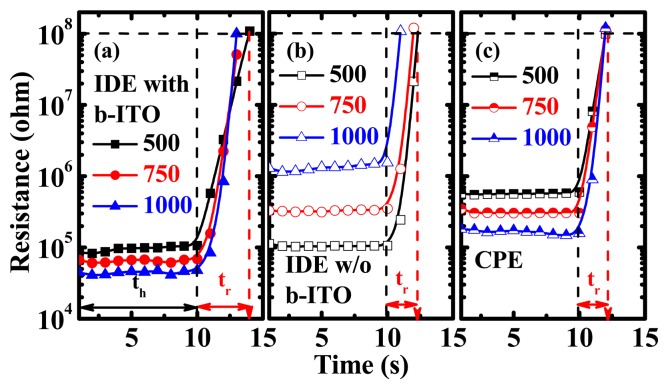
The resistance *versus* time (*R-t*) characteristics after the release of pressure of the pressure sensors with (**a**) inter-digitated electrode (IDE) with bottom indium-tin-oxide (b-ITO); (**b**) IDE without b-ITO; and (**c**) cross-point electrode (CPE) structures under a pressure of 20 kPa with a hold time of 10 s.

**Figure 9.**
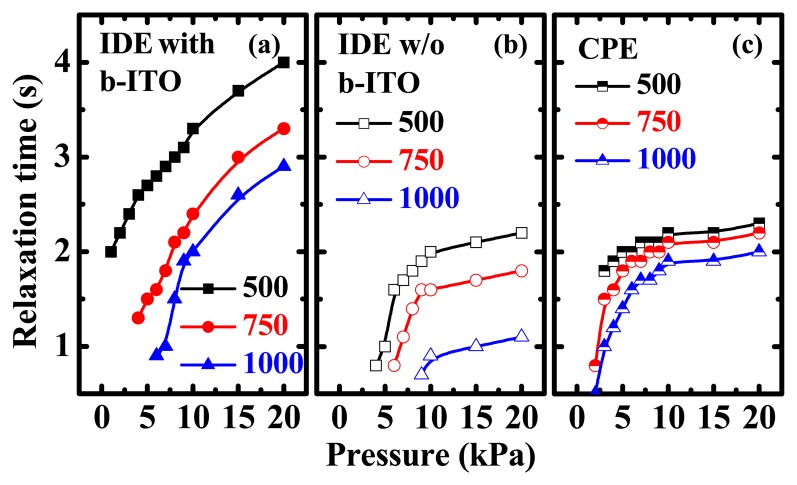
Relaxation time *versus* pressure characteristics of the pressure sensors with (**a**) inter-digitated electrode (IDE) with bottom indium-tin-oxide (b-ITO); (**b**) IDE without b-ITO; and (**c**) cross-point electrode (CPE) structures under the pressure of 0.1 to 20 kPa.

Figure 7 demonstrates the results of reversible testing of the PEDOT:PSS pressure sensors with IDE and CPE structures for at least five loops. A PEDOT:PSS spin-coating speed of 750 rpm and a pressure of 20 kPa applied under a 10-s holding time (*t_h_*) were used for the measurement. The PEDOT:PSS pressure sensors presented a stable resistive switching for two-minute sequential and reversible operations. To further investigate the response properties, the resistance *versus* time (*R-t*) characteristics after the release of pressure of the sensors are displayed in Figure 8. The relaxation time (*t_r_*) is defined as the waiting time required to reach the resistance of 100 MΩ after the normal pressure is released. It can be calculated by the following equation:

(3) $$t_{r}=t_{100\text{M}\Omega }-t_{h}$$

where *t*_100MΩ_ is the time required for the resistance to reach 100MΩ and *t_h_* is the holding time of 10 s. It can be observed that the relaxation time of the IDE pressure sensors with b-ITO is the largest. This long relaxation time is due to the combination of two carrier-conducting pathways, delaying the resistance from returning to the initial value. The relaxation times of these three pressure sensors were calculated as shown in Figure 9. It is found that when low normal pressure is applied on the pressure sensors, it produces a fast response. Furthermore, the relaxation time decreases with the film thickness, indicating that a thin PEDOT:PSS film can improve the response characteristics of pressure sensors. To obtain a fast response, it is preferable to use the structure with a single carrier conducting pathway, *i.e.*, IDE without b-ITO and CPE structures, as depicted in Figure 9b,c.

## 4. Conclusions

PEDOT:PSS piezoresistive pressure sensors with IDE and CPE structures were studied. The pressure sensors with IDE without b-ITO and with CPE structures showed a carrier conducting mechanism of horizontal and vertical pathways, which is responsible for the piezoresistive characteristic of the IDE pressure sensors with b-ITO at low and high normal pressure. With the combination of two carrier-conducting pathways, the IDE pressure sensors with b-ITO presented high piezoresistive pressure sensitivity. Besides, the decrease of resistance was observed when the normal pressure was applied because of the reduced distance between PEDOT oligomers or the increased number of conducting points within the PEDOT:PSS film. To obtain a stable reversible operation and fast piezoresistive response, a single conducting pathway of pressure sensors with IDE without b-ITO and with CPE structures can be implemented.

## Figures and Tables

**Table 1.** Summary of all parameters of the piezoresistive pressure sensors with inter-digitated electrode (IDE) with bottom indium-tin-oxide (b-ITO), without b-ITO and cross-point electrode (CPE) structures.

**Parameters**	**Structures of Piezoresistive Pressure Sensors**		

	**IDE with b-ITO**	**IDE w/o b-ITO**	**CPE**
ITO thickness (μm)	0.35	0.35	0.35
PEDOT:PSS thickness (μm)	0.87–1.88	0.87–1.88	0.87–1.88
Electrode width (μm)	500	500	3000
Electrode spacing (μm)	500	500	-
Cell area (μm^2^)	10^8^	10^8^	9 × 10^6^
